# The Combined Effect of Ear Lobe Crease and Conventional Risk Factor in the Diagnosis of Angiographically Diagnosed Coronary Artery Disease and the Short-Term Prognosis in Patients Who Underwent Coronary Stents

**DOI:** 10.1097/MD.0000000000000815

**Published:** 2015-07-02

**Authors:** Xuwei Hou, Yu Jiang, Ningfu Wang, Yun Shen, Xiaoyan Wang, Yigang Zhong, Peng Xu, Liang Zhou

**Affiliations:** From the Department of Cardiology, Hangzhou First Municipal Hospital & Hangzhou Hospital, Nanjing Medical University, Hangzhou (XH, NW, YS, XW, YZ, PX, LZ); Department of Cardiology, Second hospital of Nanchang University, Nanchang, Jiangxi, China (YJ).

## Abstract

The role of diagonal ear lobe crease (DELC) in coronary artery disease (CAD) diagnosis and prognosis remains controversial. In this study, we aimed to assess the combined effect of DELC with other conventional risk factors in the diagnosis and prognosis of CAD in Chinese patients who underwent angiography and coronary stent implantation.

The study consisted of 956 consecutive patients who underwent angiography. The DELC was identified as no DELC, unilateral, and bilateral DELC. The conventional risk factors for CAD were recorded.

Our dada showed that the overall presence of DELC is associated with CAD risk. Stratification analyses revealed that the diagnostic value of DELC was mostly significant in those with >4 risk factors. Also in patients with >4 risk factors, the presence of bilateral DELC remains to be associated with higher hs-CRP level, higher severity of CAD, and higher possibility of developing major adverse cardiac events after successful percutaneous coronary intervention (PCI).

Our study confirmed the relation of DELC with CAD in Chinese patients; more importantly, our data suggest the combination of DELC and CAD risk factors will help to predict the incidence of CAD and may predict the prognosis after successfully PCI.

## INTRODUCTION

Diagonal ear lobe crease (DELC) is a wrinkle-like line extending diagonally from the tragus across the lobule to the rear edge of the auricle of the ear.^1^ DELC is a very simple physical examination, which costs nothing and does no harm to patients. An early study in 1973 firstly showed that the presence of DELC reflects vascular atherosclerosis and is associated with the human coronary artery disease (CAD).^2^ However, the role of DELC in CAD diagnosis remains controversial because some researchers duplicated the association between DELC and CAD, whereas the others failed to do that.

The development of CAD is a multifactorial process. Epidemiological studies have documented that several factors including sex, age, smoking, hypertension, hyperlipidemia, diabetic mellitus (DM), obesity, cardiovascular disease (CVD), and family history are risk factors for CAD.^3–11^ Clinical studies showed that the accumulation of risk factors will increase the CAD risk, whereas risk factor control will reduce the CAD incidence and severity.^11,12^

As a non-specific physical sign, the solo presence of DELC may not be sufficient diagnostic marker for CAD. We hypothesize that the combination of DELC and above-mentioned conventional risk factors might be more accurate to evaluate the CAD risk. In addition, to date, little is known about prognostic role of DELC in CAD patients undergoing percutaneous coronary intervention (PCI). So the effect of DELC on the clinical outcome of PCI aroused our interest as well.

## METHODS

The study population consisted of 956 consecutive patients undergoing angiography in our hospital from January 2007 to January 2011. Definition of CAD was as described previously.^13^ All these participants were ethnically Chinese Han. Subjects with external ear disease or deformation, ear injury history, skin disease affecting ears, previous diagnosis of CAD or ischemic stroke, myocardial infarction, cardiomyopathy, and chronic diseases, such as cancer, were excluded from this study. The study protocol was approved by the Ethics Committees Ethical Committee in Hangzhou First Municipal Hospital. Written informed consent was obtained from all patients before participation in the study. Luminal stenosis was defined as significant when the luminal diameter of the major coronary artery, or one of its major branches, showed a reduction of ≥50%. The decision of coronary arteries with significant luminal stenosis was determined by 2 independent investigators by visual determination.

The severity of CAD was determined by the number of disease vessels. To gain a more accurate evaluation, we also used the Gensini scoring system to determine the CAD severity.^14^ Coronary artery score equals the sum of all segment scores (where each segment score equals segment weighting factor multiplied by severity score). Severity scores assigned to the specific percentage luminal diameter reduction of the coronary artery segment are 32 for 100%, 16 for 99%, 8 for 90%, 4 for 75%, 2 for 50%, and 1 for 25%.

The DELC was determined by checking the presence of a major crease on the earlobe on both sides. If no major crease visible on earlobe of both sides, it was assigned as “No DELC”; if only 1 major crease was observed on earlobe of 1 side, then assigned as “unilateral DELC”; if major creases were seen in both sides, then assigned as “bilateral DELC.”

Traditional risk factors for CAD, including arterial hypertension (blood pressure ≥140/90 mmHg or antihypertensive therapy), serum total cholesterol, serum high- low-density lipoprotein cholesterol (HDL-C), low-density lipoprotein cholesterol (LDL-C), triglycerides, creatinine, current or prior smokers, diabetes mellitus (DM), and a family history of CVD were recorded. The number of risk factor was recorded for each patient.

### PCI Procedure and Follow-Up

PCI was performed according to the American Heart Association/American College of Cardiology guidelines.^15^ Antegrade perfusion was graded by Thrombolysis In Myocardial Infarction^16^ criteria. All patients received drug-eluting stent. After the procedures, all patients were on dual antiplatelet therapy with aspirin and clopidogrel for one 1 year. Other treatment including statins, angiotensin-converting enzyme inhibitor, and β-blocker were given to patients. The clinical outcome was evaluated at the end of 12^th^ month. Major adverse cardiac events (MACEs) were considered as end points, including cardiac death, acute myocardial infarction (AMI), new PCI, bypass cardiac surgery, and development of congestive heart failure.^17^

### Statistical Analysis

Data on quantitative characteristics are expressed as means ± SD. Data on qualitative characteristics are expressed as percent values or absolute numbers, as indicated. Differences in demographic characteristics and vascular risk factors between patients and controls were compared by using Student *t* test or analysis of variance for continuous variables and the *χ*^2^ test for all categorical variables. The DELC frequencies in subjects with and without CAD were compared by *χ*^2^ analysis or Fisher exact test. Multivariate logistic regression analysis was used to determine the influence of DELC on CAD risk, controlling potential confounding conventional risk factors. A forward stepwise (Likelihood Ratio) procedure was used for multivariable analysis. Data were analyzed with the SAS9.3 (SAS Inc, Cary, NC). The results were considered statistically significant at *P* < 0.01 using a 2-tailed test.

## RESULTS

Based on the results of angiography, 446 subjects were assigned to the CAD group, and 510 cases were assigned to non-CAD group. Demographic characteristics of CAD groups and non-CAD groups are presented in Table 1. The CAD group had older age, higher systolic blood pressure level, higher BMI, LDL, CRP level, had higher percentage of male, DM, smoker, and CVD family history than non-CAD group (All *P* < 0.05).

**TABLE 1:**
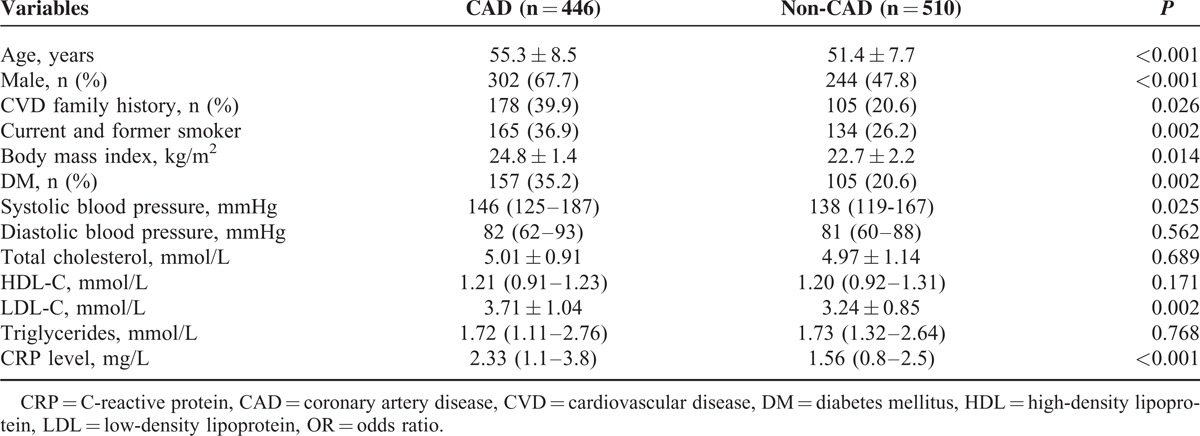
Demographic Characteristics of All Participants

Table 2 shows the comparison of overall DELC presence between CAD and non-CAD groups. Our data showed there is a significant difference in the overall DELC appearance between the 2 groups. The CAD group had markedly increased frequencies of bilateral DELC compared with non-CAD group (39% vs 27%, *P* < 0.001). To determine whether DELC is associated with the CAD risk, we performed the logistic regression analyses. Our results showed that the presence of bilateral DELC represented a significantly higher risk for CAD with adjustment with age, sex, BMI, blood pressure, LDL, smoking status, DM, and family history of CVD (adjusted odds ratio [OR] = 1.917, compared with no DELC subgroup, adjusted *P* < 0.001). However, the unilateral presence of DELC was not associated with CAD risk (*P* = 0.183).

**TABLE 2:**
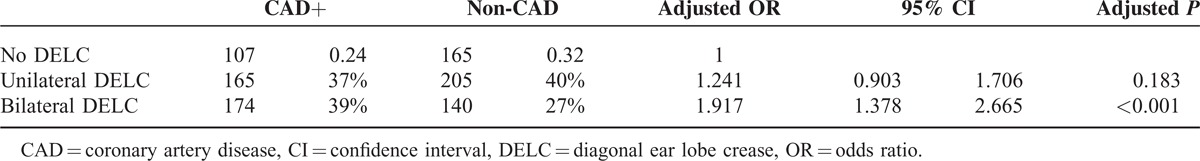
The Comparison of Overall DELC Presence Between CAD and Non-CAD Groups

To analyze the interaction between DELC and conventional risk factors for CAD, we next stratified the enrolled subjects by the numbers of risk factors each patient carried. Table 3 shows the frequencies of DELC in each stratification subgroups. In those with <3 risk factors, the DELC frequencies were similar between CAD and non-CAD groups (*P* = 0.303). In patients with 3 to 4 risk factors, a significant difference in DELC frequencies was noted (*P* = 0.039). In this subgroup, those with bilateral DELC had 1.882 times higher chance to develop CAD (95% confidence interval [CI]: 1.089–3.253, *P* = 0.023). A significant difference in DELC frequencies was noted more evident in those with >4 risk factors (*P* < 0.001). The presence of bilateral DELC in this subgroup represented 4.047 times higher chance to have CAD (95% CI: 2.267–7.226, *P* < 0.001). Unilateral DELC was not associated with the CAD risk in all subgroups.

**TABLE 3:**
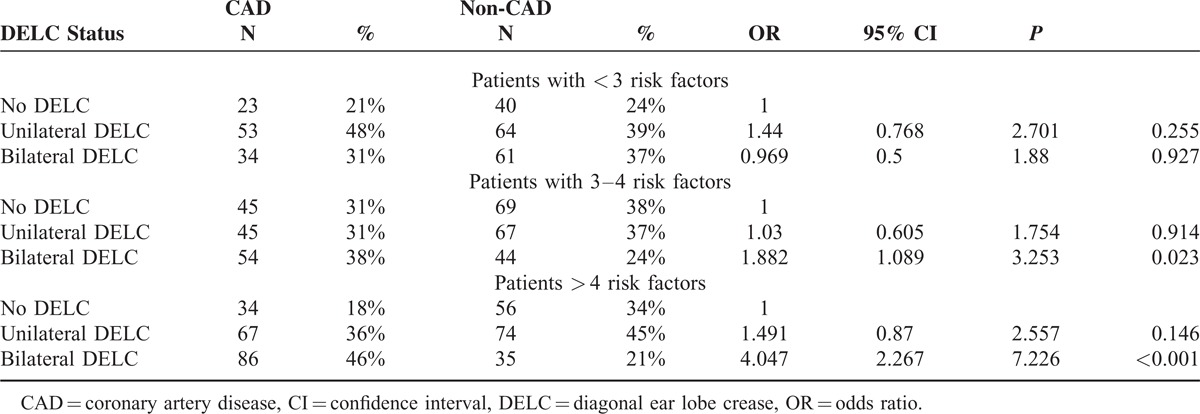
The Interaction Between DELC and the Number of Conventional Risk Factors for CAD

We next analyzed the association of DELC with the severity of CAD by the number of diseased vessels and by the Gensini scores. We found that the overall DELC appearance did not affect the number of disease vessels (Figure 1A) and Gensini scores (Figure 1B). There was no significant difference in the disease severity among patients with <3 or 3 to 4 risk factors. Only among the patients with >4 risk factors, there is a significant increase in the diseased vessel numbers (Figure 1A–D) and Gensini scores (Figure 1B–D) among patients with bilateral DELC, compared with those without DELC and those with unilateral DELC. The bilateral DELC carriers had significantly higher diseased number than those without or with only unilateral DELC (2.3 ± 0.2 vs 1.7 ± 0.1, *P* = 0.013; vs 1.8 ± 0.1, *P* = 0.021 Figure 1D). Likewise, patients with bilateral DELC had markedly higher Gensini scores (78.3 ± 10.6) than those without DELC (47.3 ± 11.2, *P* = 0.006) and those with unilateral DELC (58.1 ± 8.6, *P* = 0.010).

**FIGURE 1:**
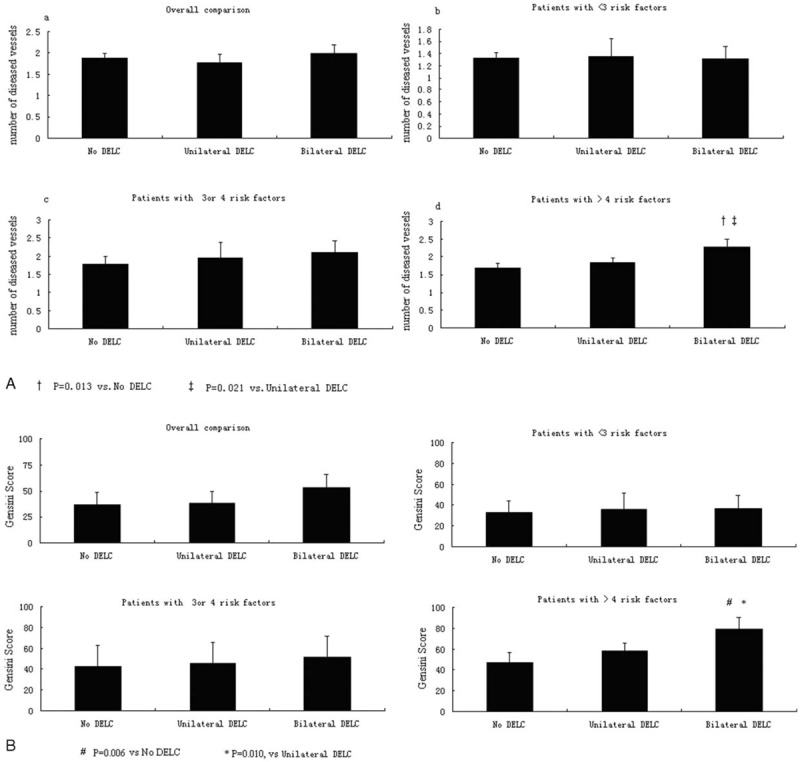
The association of diagonal ear lobe crease (DELC) with the coronary artery disease (CAD) severity. (A) The association of DELC with the CAD severity determined by the number of diseased vessels. (B) The association of DELC with the CAD severity determined by Gensini scores. Those patients with bilateral DELC had significantly higher severity of CAD compared with those without DELC and with unilateral DELC.

The associations of DELC presence with blood pressure, serum HDL-C, LDL-C level, and CRP at diagnosis were also studied. We found that the mean levels of these variables were similar among patients without DELC, unilateral, and bilateral DELC, even after stratification by the number of risk factors (data not shown). As for the serum CRP levels, the mean CRP level was higher in those with bilateral DELC compared with those without DELC, but did not reach significant difference (*P* = 0.052, Figure 2A). After stratification with the number of risk factors, we observed that patients with bilateral DELC from subgroup with >4 risk factors had a significantly higher mean CRP level (2.45 [2.08–2.51] mg/L) than those without (1.65 [1.45–2.05] mg/L, *P* = 0.014) or only with unilateral DELC (1.92 [1.64—2.2] mg/L, *P* = 0.016) (Figure 2D). In patient with <3 and with 3 to 4 risks, the CRP levels were not noted significantly different (both *P* > 0.05, Figure 2B and C).

**FIGURE 2:**
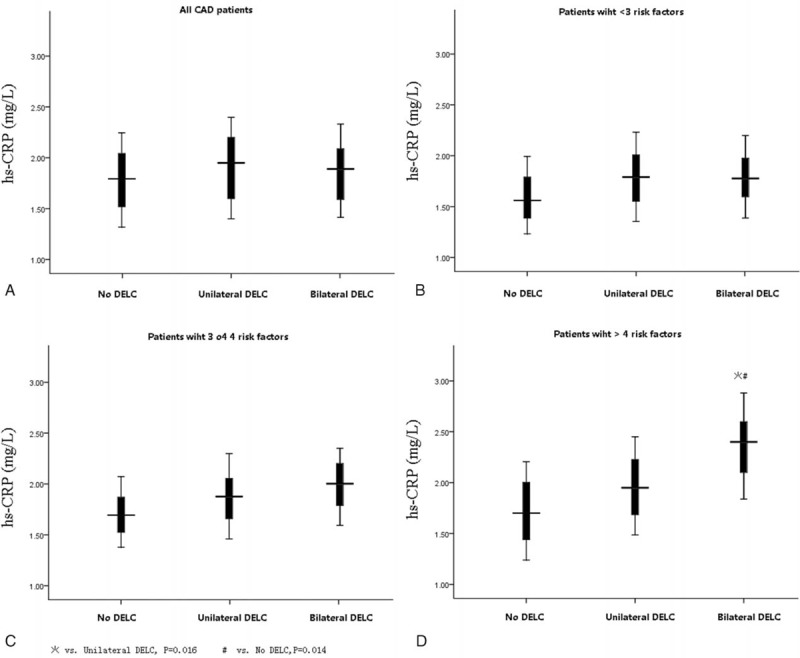
The mean C-reactive protein (CRP) level among patients without diagonal ear lobe crease (DELC), unilateral DELC, and bilateral DELC stratified by the number of risk factors.

Of all CAD patients, there were 276 patients who underwent successful PCI and were follow-up for 12 month. Of all patients, 17 had major adverse cardiac event (MACE) during follow-up period (6.15%). The overall DELC frequencies between patients with MACE and those without MACE were not significantly different (*P* = 0.155, Table 4). Logistic regression analyses with conventional risk factors did not reveal any association of DELC, both unilateral and bilateral, with MACE incidence. However, when we stratified these patients according to the number of risk factors each one carried, we found that in only patients with >4 risk factors, the presence of bilateral DELC represented a significantly increased risk for MACE incidence (OR = 5.568, *P* = 0.028, Table 5).

**TABLE 4:**
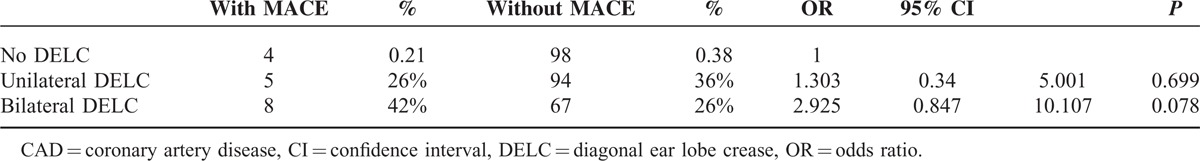
The Overall DELC Frequencies Between Patients With and Without MACE

**TABLE 5:**
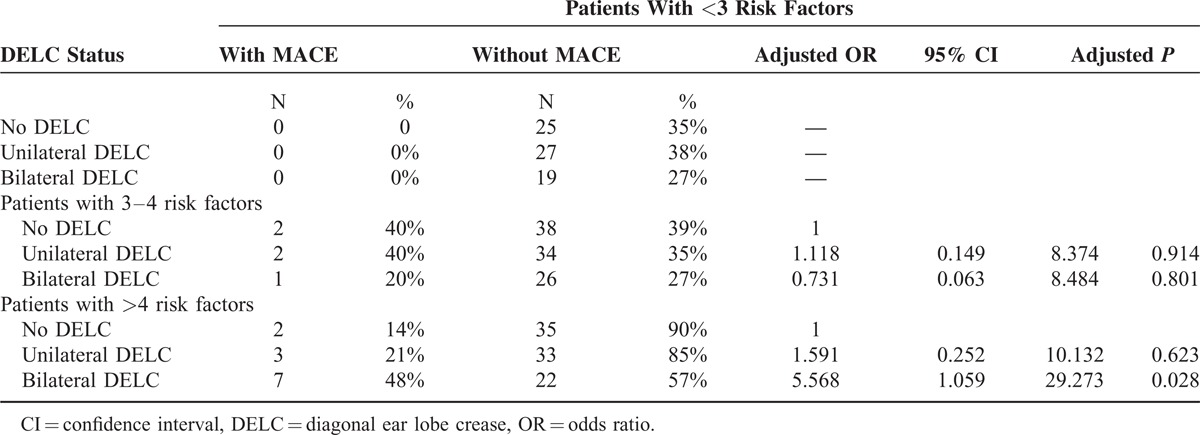
The DELC Frequencies Between Patients With and Without MACE Stratified by the Number of Risk Factors

To exclude that the worse prognosis after PCI in the presence of DELC is due to a worse atherosclerotic coronary artery disease, we stratified all patients based on their Gensini scores (<30, 31–59, and >60). Similarly, we found that the DELC presence was not associated with the MACE in patients with Gensini scores <30 and between 31 and 59; however, in those with Gensini scores >60, the bilateral DELC had dramatically increased chance to develop MACE (OR = 6.002, *P* = 0.019, Table 6).

**TABLE 6:**
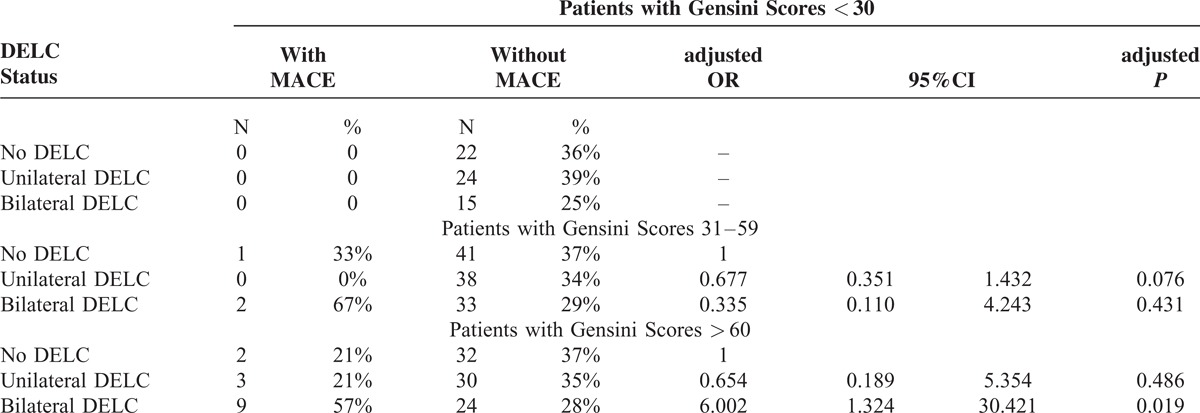
The DELC frequencies between patients with and without MACE stratified by Gensini Scores

## DISCUSSION

In this study, we emphasized the interaction between DELC presence and the conventional risk factors in the diagnosis of angiographically determined CAD and the clinical outcome in patients who underwent successful stent procedures. Our data showed that the overall presence of DELC is associated with CAD incidence. Stratification analyses revealed that the diagnostic value of DELC was mostly significant in those with >4 risk factors. DELC presence along did not predict the MACE incidence after PCI; however, in patients with >4 risk factors, the presence of bilateral DELC remains to be associated with higher chance of developing MACE after successful PCI. This result confirmed a positive association between DELC and CAD risk, in addition, suggesting that the combination of DELC with risk factor each individual carriers will increase its diagnostic value in predicting the CAD and its outcome after PCI.

The association between DELC and ELC has been reported in several independent studies. DELC (both unilateral or bilateral) was significantly more common among American patients with an AMI than among age-matched control subjects.^18^ In Indian population, bilateral DELC was found to be significantly associated with angiographically documented CAD.^19^ In Irish patients, the asassociation between an DELC and CAD was found to be significant, suggesting that it should be used as a physical sign predictive of the presence of coronary heart disease.^20^ DELC was reported more prevalent in male patients than female patients and the incidence of bilateral presence of ELC showed an age-related increase trend.^21^ In a Japanese population, patients with ELC had significantly higher carotid intima thickness than controls.^22^ A very recent study in the United States revealed that the DELC was independently and significantly associated with increased prevalence, extent, and severity of CAD defined by computed tomography angiography.^23^

However, most of researchers studied overall effect of DELC on CAD risk. Little is known about the combined effect of the DELC and the conventional CAD risk factors on CAD. Our study provides evidence that there is also a positive association between DELC with the incidence of Chinese CAD defined by angiography.

One novelty of this study is that we analyzed the combined effect of DELC presence and the number of risk factors on CAD risk. As far as we know, no similar study was reported. Our data suggest the combination of DELC and conventional risk factors has increased value in predicting the outcome of CAD patients receiving PCI.

Another novelty of this study is that we investigated the role of DELC in the clinical outcome of patients who underwent successful coronary artery drug-eluting stents implantation. An autopsy study showed that ELC was one of the strongest independent risk factor for sudden cardiac death.^24^ Another follow-up study showed that a diagonal ELC is associated with increased all-cause and cardiac morbidity and mortality. Patients with ELC may be at higher risk for coronary events, even if currently without diagnostic evidence of CAD.^25^ Currently, there is no studying reporting the diagnostic role of DELC in patients underwent PCI. In this study, we found that in patients with >4 risk factors, the presence of bilateral DELC remains to be associated with higher chance of developing MACE after successful PCI.

## CONCLUSION

Taken together, our study confirmed the relation of DELC with CAD in Chinese patients; moreover, our data show that the value of DELC is more reliable in those with multiple risk factors, suggesting the importance of combination of DELC and CAD risk factors.
